# Hospitalizations for fluid overload and heart failure among individuals with diabetes: healthcare utilization and guideline-directed medical therapy

**DOI:** 10.1093/ckj/sfae005

**Published:** 2024-01-08

**Authors:** Zhihua Huang, Ee Won Leong, Lourdes Ducusin Galang, Li Choo Ng, Felicia Loo, Lydia Lim, Hanis Binte Abdul Kadir, Cynthia Ciwei Lim

**Affiliations:** Department of Renal Medicine, Singapore General Hospital, Singapore, Singapore; Specialty Nursing, Singapore General Hospital, Singapore, Singapore; Specialty Nursing, Singapore General Hospital, Singapore, Singapore; Department of Internal Medicine, Singapore General Hospital, Singapore, Singapore; Department of Internal Medicine, Singapore General Hospital, Singapore, Singapore; Department of Renal Medicine, Singapore General Hospital, Singapore, Singapore; Specialty Nursing, Singapore General Hospital, Singapore, Singapore; Department of Renal Medicine, Singapore General Hospital, Singapore, Singapore; Department of Renal Medicine, Singapore General Hospital, Singapore, Singapore; Health Service Research Unit, Singapore General Hospital, Singapore, Singapore; Department of Renal Medicine, Singapore General Hospital, Singapore, Singapore

To the Editor,

In ‘Practical approaches to building up a cardiorenal clinic’ by Espriella *et al*., the authors recognized the burgeoning population with concomitant cardiovascular and kidney disease which will require significant healthcare resources [1]. Such cardiorenal disease is prevalent in diabetes. Diabetes causes macrovascular and microvascular disease in the heart and kidney. Additionally, interactions between deranged organ systems can further exacerbate cardiovascular and kidney dysfunction bidirectionally, and result in cardiorenal syndrome. Common complications of the cardiorenal syndrome are kidney sodium avidity and fluid retention which may manifest as fluid overload and/or decompensated heart failure [2], and differentiating these conditions in kidney disease remains challenging [1, 2].

To quantify the healthcare utilization and understand the clinical profile of patients with these conditions, we examined the hospitalizations for fluid overload and heart failure among individuals with diabetes in the SingHealth Diabetes Registry between 2018 and 2021. The International Classification of Diseases 10th Revision codes E877, I50, I500, J81 and R601 were used to identify hospitalizations for the diagnoses of fluid overload, heart failure, congestive heart failure, pulmonary edema and generalized edema. Among 24 310 hospitalizations, 71.9% were older adults aged ≥65 years. Cardiovascular disease and chronic kidney disease (CKD; estimated glomerular filtration rate <60 mL/min/1.73 m^2^ at admission) were very common, at 85.0% and 68.7%, respectively. The diagnosis code for fluid overload (64.4%) was more frequently identified than the codes for heart failure or congestive heart failure (40.5%). The majority (89.2%) were admitted via the emergency department and the mean (± standard deviation) length of stay was 10.8 ± 16.5 days. Over a follow-up of median 14.7 (interquartile range 3.4, 28.4) months, 50.0% were readmitted for recurrence of fluid overload or heart failure at 2.9 (0.8, 9.1) months after discharge. The number of hospitalizations had increased from an average of 424.4 hospitalizations per month in the period 2018–19 to 588.5 hospitalizations per month in 2020–21. The data presented in Table 1 suggest that an older population and a greater burden of comorbidities, including metabolic risk factors and more severe CKD, may be driving the increased hospitalizations. Yet, only 84.2% and 66.9% of the hospitalizations for fluid overload and heart failure had glycated hemoglobin (HbA1c) and lipid evaluation during or within 6 months before the hospitalization. The mean (± standard deviation) HbA1c was 7.3% ± 1.7%, while the total cholesterol, low-density lipoprotein cholesterol and high-density lipoprotein cholesterol were 3.87 ± 1.40, 2.17 ± 1.14 and 1.14 ± 0.36 mmol/L, respectively. Table 1 shows that the use of pharmacotherapy such as renin–angiotensin system (RAS) blockers, sodium-glucose co-transporter 2 (SGLT2) inhibitors, glucagon-like peptide 1 (GLP1) agonists and statins before hospitalization and at discharge were low compared with the high prevalence of cardiovascular and kidney disease in this cohort. These results may reflect the challenges in implementing evidence-based best practices [3].

**Table 1:** Hospitalizations for fluid overload and heart failure among individuals with diabetes.

	Total	2018–19	2020–21	
	*N* = 24 310	*N* = 10 186	*N* = 14 124	*P*-value^a^
Clinical profile				
Male, *n* (%)	13 172 (54.2)	5540 (54.4)	7632 (54.0)	.59
Age ≥65 years, *n* (%)	17 472 (71.9)	7158 (70.3)	10 314 (73.0)	<.001
Charlson Comorbidity Index ≥7, *n* (%)	19 782 (81.4)	7868 (77.2)	11 914 (84.4)	<.001
Hypertension, *n* (%)	22 607 (93.0)	9381 (92.1)	13 226 (93.6)	<.001
CKD, *n* (%)	16 692 (68.7)	6834 (67.5)	9858 (70.2)	<.001
Stage G3 and G4	11 777 (48.4)	5119 (50.3)	6658 (47.1)	<.001
Stage G5	4915 (20.2)	1715 (16.9)	3200 (22.8)	<.001
Cardiovascular disease, *n* (%)	20 670 (85.0)	8789 (96.3)	11 881 (84.1)	<.001
Atrial fibrillation, *n* (%)	8048 (33.1)	3363 (33.0)	4685 (33.2)	.80
Cancer, *n* (%)	2730 (11.2)	990 (9.7)	1740 (12.3)	<.001
Pharmacotherapy				
RAS blocker, *n* (%)				
Pre-hospitalization	8869 (36.5)	4090 (40.2)	4779 (33.8)	<.001
At discharge	12 881 (53.0)	5939 (58.3)	6942 (49.2)	<.001
SGLT2 inhibitor, *n* (%)				
Pre-hospitalization	1380 (5.7)	367 (3.6)	1013 (7.2)	<.001
At discharge	3326 (13.7)	871 (8.6)	2455 (17.4)	<.001
GLP1 agonist, *n* (%)				
Pre-hospitalization	55 (0.2)	17 (0.2)	38 (0.3)	.1
At discharge	109 (0.4)	24 (0.2)	85 (0.6)	<.001
Statin, *n* (%)				
Pre-hospitalization	13 876 (57.1)	5926 (58.2)	7950 (56.3)	.003
At discharge	20 789 (85.5)	8719 (85.6)	12 070 (85.5)	.76

^a^Comparison between the time periods of 2018–19 and 2020–21: categorical variables were presented as proportions and compared using Pearson chi-square test.

**Figure 1:**
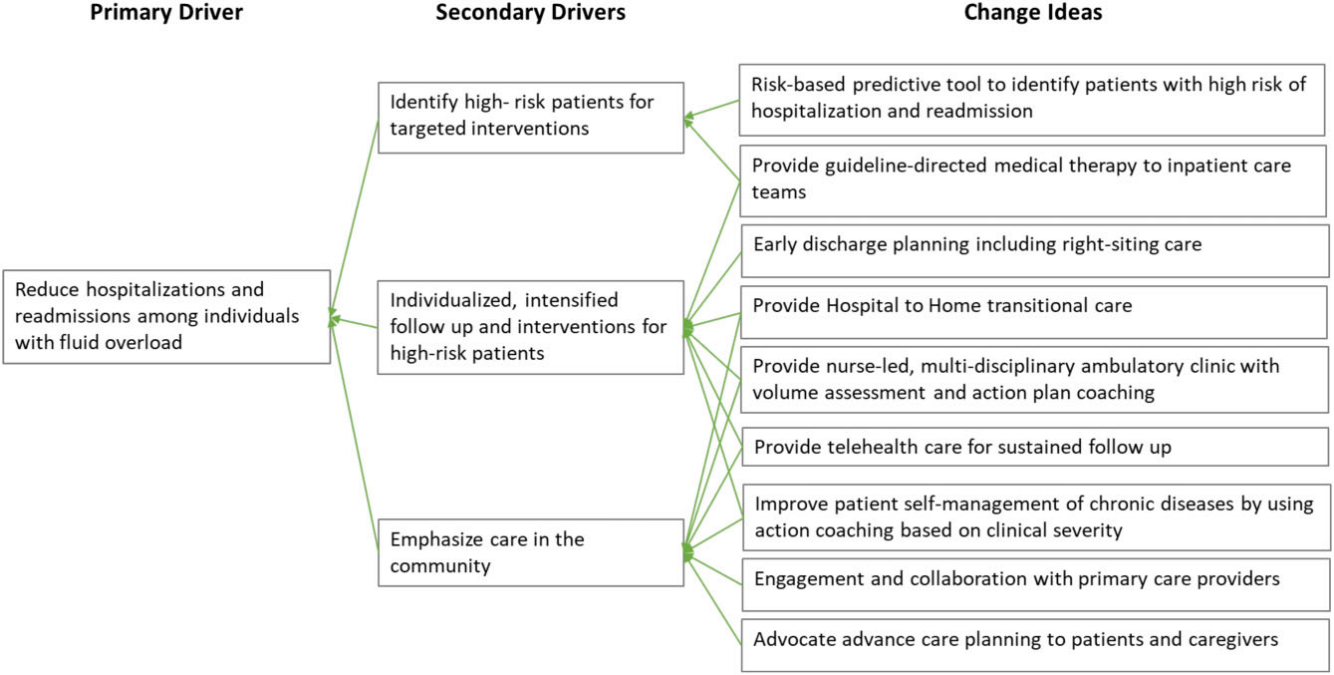
Driver diagram for interventions to reduce hospitalizations and readmissions for fluid overload.

Metabolic risk factor control and pharmacotherapy can reduce cardiovascular and kidney disease progression risk [4], and therefore reduce hospitalizations related to the cardiorenal syndrome. A systematic review of 13 trials of more than 90 000 participants noted that SGLT2 inhibitors reduced the risk of cardiovascular death and hospitalization for heart failure and kidney disease progression in diabetes [5]. Both the American Diabetes Association and Kidney Disease: Improving Global Outcomes recommend a SGLT2 inhibitor as first-line pharmacotherapy in diabetes and CKD with estimated glomerular filtration rate ≥20 mL/min/1.73 m^2^, in addition to the long-established RAS blocker [4]. Additionally, a statin is also recommended at moderate to high intensity according to the individual's cardiovascular risk, while a GLP1 agonist is recommended as add-on therapy to achieve glycemic control [4]. Therefore, there is a need to optimize guideline-directed medical therapy (GDMT) that reduces cardiovascular and kidney disease risks in diabetes. To address the possible barriers of low awareness or knowledge among physicians and patients, clinical inertia and fragmented care [3], we piloted strategies (Fig. 1) that emphasized the identification of high-risk patients, communication of GDMT to the inpatient care teams, patient education and action plan coaching, and intensified follow-up that was individualized according to patients’ preferences, presence of multi-morbidity and ability to access community care. The cardiorenal program implemented by Espriella *et al*. similarly emphasized optimization of pharmacological treatment and patient-centered care in transitioning between different levels of care [1]. In addition to the outcome and process indicators described, cardiorenal programs should also use patient-reported outcome measures related to health literacy, self-management and health-related quality of life to reflect the quality of patient-centered care.
